# The cocrystal aqua­chlorido{6,6′-di-*tert*-butyl-2,2′-[1,2-phenyl­enebis(nitrilo­methyl­idyne)]diphenolato-κ^4^ *O*,*N*,*N*′,*O*′}­manganese(III)–chlorido{6,6′-di-*tert*-butyl-2,2′-[1,2-phenyl­enebis(nitrilo­methyl­idyne)]diphenolato-κ^4^ *O*,*N*,*N*′,*O*′}(methanol-κ*O*)manganese(III) (1/1)

**DOI:** 10.1107/S1600536808006818

**Published:** 2008-04-04

**Authors:** Naser Eltaher Eltayeb, Siang Guan Teoh, Suchada Chantrapromma, Hoong-Kun Fun, Rohana Adnan

**Affiliations:** a School of Chemical Science, Universiti Sains Malaysia, 11800 USM, Penang, Malaysia; b Department of Chemistry, Faculty of Science, Prince of Songkla University, Hat-Yai, Songkhla 90112, Thailand; c X-ray Crystallography Unit, School of Physics, Universiti Sains Malaysia, 11800 USM, Penang, Malaysia

## Abstract

The asymmetric unit of the title complex, [Mn(C_28_H_30_N_2_O_2_)Cl(H_2_O)][Mn(C_28_H_30_N_2_O_2_)Cl(CH_3_OH)], contains two discrete Mn^III^ complexes of a Schiff base ligand, with an N_2_O_2_ donor set. Both Mn^III^ centers are in a distorted octa­hedral geometry with the N_2_O_2_ donor atoms of the tetra­dentate Schiff base dianion in the equatorial plane. The axial positions in the coordination environment of one Mn^III^ complex are occupied by a chloride ion and a water mol­ecule, but a methanol mol­ecule replaces the water mol­ecule in the other complex. The coordinated water mol­ecule takes part in an O—H⋯Cl hydrogen bond between the two Mn^III^ complexes. In the crystal structure, O—H⋯Cl hydrogen bonds link the mol­ecules into infinite one-dimensional chains along the [100] direction. The crystal structure is stabilized by O—H⋯Cl hydrogen bonds together with weak C—H⋯O and C—H⋯Cl inter­actions. A C—H⋯π inter­action is also observed in the crystal structure.

## Related literature

For bond-length data, see: Allen *et al.* (1987 ▶). For related structures, see for example: Eltayeb *et al.* (2007 ▶, 2008 ▶); Habibi *et al.* (2007 ▶); Mitra *et al.* (2006 ▶). For background to applications of manganese complexes, see for example: Dixit & Srinivasan (1988 ▶); Glatzel *et al.* (2004 ▶); Lu *et al.* (2006 ▶); Stallings *et al.* (1985 ▶).
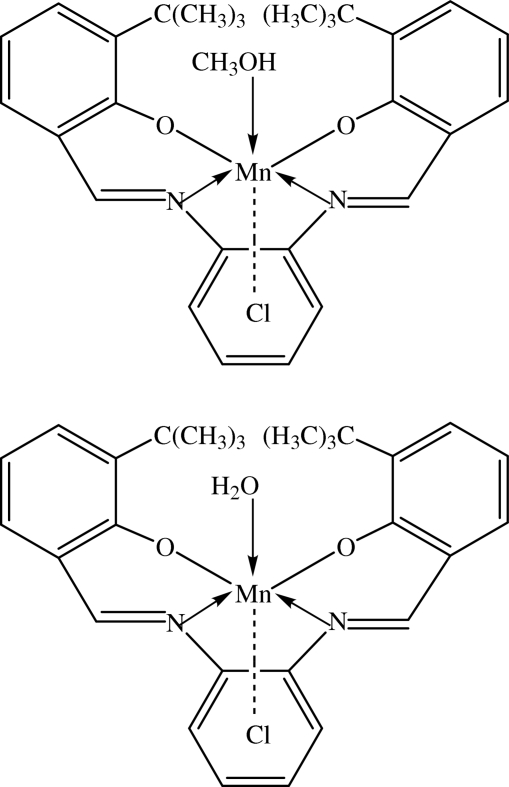


## Experimental

### 

#### Crystal data

[Mn(C_28_H_30_N_2_O_2_)Cl(H_2_O)][Mn(C_28_H_30_N_2_O_2_)Cl(CH_4_O)]
*M* *_r_* = 1083.92
Triclinic, 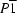

*a* = 13.1080 (3) Å
*b* = 13.8794 (3) Å
*c* = 14.6085 (3) Å
α = 95.177 (1)°
β = 99.996 (1)°
γ = 95.639 (1)°
*V* = 2589.08 (10) Å^3^
*Z* = 2
Mo *K*α radiation
μ = 0.65 mm^−1^
*T* = 100.0 (1) K
0.38 × 0.33 × 0.03 mm

#### Data collection

Bruker SMART APEX2 CCD area-detector diffractometer
Absorption correction: multi-scan (*SADABS*; Bruker, 2005 ▶) *T*_min_ = 0.791, *T*_max_ = 0.981
37620 measured reflections
10108 independent reflections
6163 reflections with *I* > 2σ(*I*)
*R*_int_ = 0.076

#### Refinement

*R*[*F* ^2^ > 2σ(*F* ^2^)] = 0.057
*wR*(*F* ^2^) = 0.147
*S* = 1.03
10108 reflections
653 parameters
H-atom parameters constrained
Δρ_max_ = 0.56 e Å^−3^
Δρ_min_ = −0.52 e Å^−3^

### 

Data collection: *APEX2* (Bruker, 2005 ▶); cell refinement: *APEX2*; data reduction: *SAINT* (Bruker, 2005 ▶); program(s) used to solve structure: *SHELXTL* (Sheldrick, 2008 ▶); program(s) used to refine structure: *SHELXTL*; molecular graphics: *SHELXTL*; software used to prepare material for publication: *SHELXTL* and *PLATON* (Spek, 2003 ▶).

## Supplementary Material

Crystal structure: contains datablocks global, I. DOI: 10.1107/S1600536808006818/sj2472sup1.cif

Structure factors: contains datablocks I. DOI: 10.1107/S1600536808006818/sj2472Isup2.hkl

Additional supplementary materials:  crystallographic information; 3D view; checkCIF report

## Figures and Tables

**Table 1:** Hydrogen-bond geometry (Å, °)

*D*—H⋯*A*	*D*—H	H⋯*A*	*D*⋯*A*	*D*—H⋯*A*
O1*WA*—H2*WA*⋯Cl1*B*	0.85	2.28	3.113 (3)	167
O3*B*—H1*O*3⋯Cl1*A*^i^	1.00	2.06	3.026 (3)	163
C5*A*—H5*AA*⋯O1*WA*^ii^	0.93	2.55	3.463 (5)	169
C4*B*—H4*BA*⋯Cl1*A*^ii^	0.93	2.79	3.528 (4)	137
C12*B*—H12*B*⋯Cl1*A*^iii^	0.93	2.73	3.646 (4)	170
C23*A*—H23*C*⋯O1*A*	0.96	2.34	2.984 (6)	124
C23*B*—H23*E*⋯O1*B*	0.96	2.35	2.983 (5)	123
C24*A*—H24*C*⋯O1*A*	0.96	2.34	2.975 (5)	123
C24*B*—H24*E*⋯O1*B*	0.96	2.36	3.010 (5)	124
C26*A*—H26*A*⋯O2*A*	0.96	2.45	3.041 (5)	119
C26*B*—H26*E*⋯O2*B*	0.96	2.35	2.998 (5)	124
C28*A*—H28*A*⋯O2*A*	0.96	2.34	2.977 (5)	124
C28*B*—H28*F*⋯O2*B*	0.96	2.34	2.968 (5)	122
C14*B*—H14*B*⋯*Cg*1^iv^	0.93	3.23	3.690 (4)	113

Symmetry codes: (i) 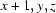; (ii) 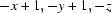; (iii) 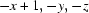; (iv) 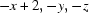. *Cg*1 is the centroid of the C8*B*–C13*B* benzene ring.

**Fig. 1.**
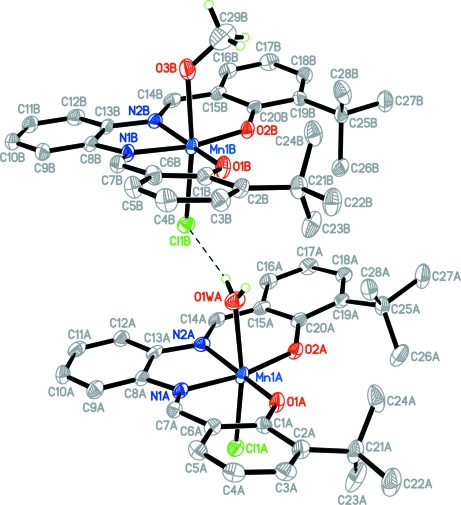
The asymmetric unit of (I), showing 50% probability displacement ellipsoids and the atomic numbering. H atoms of the Schiff base ligand were omitted for clarify. The O—H···Cl hydrogen bond is drawn as a dashed line.

**Fig. 2.**
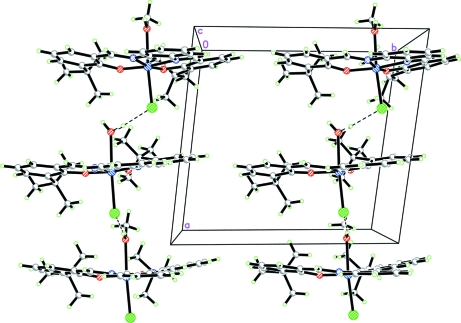
The crystal packing of (I), viewed along the c axis showing the chains running along the [1 0 0] direction. Hydrogen bonds are drawn as dashed lines.

## supplementary crystallographic information

### Comment

Manganese complexes with Schiff base ligands have been of interest due to the variety of their applications in coordination chemistry, physics, catalysis and biological replication. They have been used as models for the oxygen-evolving complex of photosystem II (Glatzel *et al.*, 2004), in catalysis (Dixit & Srinivasan, 1988), as single-molecule magnets (Lu *et al.*, 2006) and serve as models for the active sites of manganese-containing metal enzymes (Stallings *et al.*, 1985). We have previously reported the crystal structure of manganese complexes with Schiff base ligands containing oxygen and imine nitrogen atoms (Eltayeb *et al.*, 2007; 2008). In this paper, we report the crystal structure of a Mn(III) complex of the closely related ligand {6,6'-di-*tert*-butyl-2,2'-[1,2-phenylenebis(nitrilomethylidene)]diphenolate.

The asymmetric unit of the title complex molecule (Fig. 1) contains two Mn^III^ complexes (*A* and *B*) with the same Schiff base ligand. Coordination spheres around Mn^III^ in both *A* and *B* are slightly distorted octahedra, with the coordination plane of each Mn^III^ formed by the N_2_O_2_ donor atoms of the Schiff base. The axial positions in *A* are occupied by a Cl^-^ ion and a water molecule whereas in *B*, these positions are occupied by a Cl^-^ ion and a CH_3_OH molecule. The in-plane Mn—O distances are in the range 1.863 (2)–1.882 (2) Å with Mn—N distances 1.978 (3)–1.997 (3) Å, which fall in the range observed for six other Mn^III^ coordination complexes of Schiff base ligands (Eltayeb *et al.*, 2007; 2008; Habibi *et al.*, 2007; Mitra *et al.*, 2006). The elongation of the Mn—O and Mn—Cl axial bonds [2.402 (3) and 2.5420 (12) Å in *A* and 2.293 (3) and 2.5416 (11) Å in *B*] clearly indicate the usual Jahn Teller distortion of the Mn^III^ oxidation state as has been found previously (Eltayeb *et al.*, 2007; 2008; Habibi *et al.*, 2007; Mitra *et al.*, 2006). The basal bond angles O–Mn–O and O–Mn–N are close to 90° whereas the N–Mn–N angles are less than 90° [N1A–Mn1A–N2A = 82.19 (12)° and N1B–Mn1B–N2B = 81.67 (12)°]. The axial bond angle Cl–Mn–O is also less than the ideal value of 180° [170.55 (7)° in *A* and 171.74 (8)° in *B*]. Other bond lengths and angles observed in the structure are also normal (Allen *et al.*, 1987). The coordinated water molecule of molecule *A* forms an O—H···Cl hydrogen bond with the coordinated Cl^-^ ion of molecule *B* (Fig. 1). The dihedral angles between the two outer phenolate rings [(C1–C6) and C15–C20) of the tetradentate Schiff base ligand is 22.21 (19)° in *A* and 18.81 (19)° in *B*°. The central benzene ring (C8–C13) makes dihedral angles of 13.43 (19)° and 8.79 (19)° with the two outer phenolate rings in *A* [10.94° and 10.37 (19)° in *B*].

In the crystal structure (Fig. 2), O—H···Cl hydrogen bonds [O1WA—H2WA···Cl1B; symmetry code *x*, *y*, *z* and O3B—H1O3—Cl1A; symmetry code 1 + *x*, *y*, *z*) (Table 1)] link the Mn^III^ complex molecules into infinite one-dimensional chains along the [1 0 0] direction. The crystal is stabilized by these O—H···Cl hydrogen bonds, together with weak C—H···O and C—H···Cl interactions and further stabilized by C—H···π interactions (Table 1); *Cg*_1_ is the centroid of the C8B–C13B benzene ring.

### Experimental

The title compound was synthesized by adding 3-*tert*-butyl-2-hydroxybenzaldehyde (0.72 ml, 4 mmol) to a solution of *o*-phenylenediamine (0.216 g, 2 mmol) in ethanol 95% (30 ml). The mixture was refluxed with stirring for half an hour. Manganese chloride tetrahydrate (0.394 g, 2 mmol) in ethanol (10 ml) was then added, followed by triethylamine (0.5 ml, 3.6 mmol). The mixture was refluxed at room temperature for three hours. A brown precipitate was obtained, washed with about 5 ml e thanol, dried, and then washed with copious quantities of diethylether. Brown single crystals of the title compound suitable for *x*-ray structure determination were recrystallized from methanol/acetone (2:1 *v*/*v*) by slow evaporation of the solvent at room temperature after three weeks.

### Refinement

All H atoms were placed in calculated positions with d(O—H) = 0.85 Å, *U*_iso_=1.2*U*_eq_, d(C—H) = 0.93 Å, *U*_iso_=1.2*U*_eq_(C) for CH and aromatic, 0.96 Å, *U*_iso_ = 1.5*U*_eq_(C) for CH_3_ atoms. A rotating group model was used for the methyl groups. The highest residual electron density peak is located at 1.00 Å from Mn1B and the deepest hole is located at 0.85 Å from Mn1A.

### Crystal data

**Table:**

[Mn(C_28_H_30_N_2_O_2_)Cl(H_2_O)][Mn(C_28_H_30_N_2_O_2_)Cl(CH_4_O)]	*Z* = 2
*M**_r_* = 1083.92	*F*_000_ = 1136
Triclinic, *P*1	*D*_x_ = 1.390 Mg m^−^^3^
Hall symbol: -P 1	Mo *K*α radiation λ = 0.71073 Å
*a* = 13.1080 (3) Å	Cell parameters from 10108 reflections
*b* = 13.8794 (3) Å	θ = 1.6–26.0º
*c* = 14.6085 (3) Å	µ = 0.65 mm^−^^1^
α = 95.177 (1)º	*T* = 100.0 (1) K
β = 99.996 (1)º	Plate, brown
γ = 95.639 (1)º	0.38 × 0.33 × 0.03 mm
*V* = 2589.08 (10) Å^3^	

### Data collection

**Table:**

Bruker SMART APEX2 CCD area-detector diffractometer	10108 independent reflections
Radiation source: fine-focus sealed tube	6163 reflections with *I* > 2σ(*I*)
Monochromator: graphite	*R*_int_ = 0.076
Detector resolution: 8.33 pixels mm^-1^	θ_max_ = 26.0º
*T* = 100.0(1) K	θ_min_ = 1.6º
ω scans	*h* = −16→15
Absorption correction: multi-scan(SADABS; Bruker, 2005)	*k* = −17→17
*T*_min_ = 0.791, *T*_max_ = 0.981	*l* = −18→18
37620 measured reflections	

### Refinement

**Table:**

Refinement on *F*^2^	Secondary atom site location: difference Fourier map
Least-squares matrix: full	Hydrogen site location: inferred from neighbouring sites
*R*[*F*^2^ > 2σ(*F*^2^)] = 0.057	H-atom parameters constrained
*wR*(*F*^2^) = 0.147	*w* = 1/[σ^2^(*F*_o_^2^) + (0.069*P*)^2^ + 0.4229*P*] where *P* = (*F*_o_^2^ + 2*F*_c_^2^)/3
*S* = 1.03	(Δ/σ)_max_ < 0.001
10108 reflections	Δρ_max_ = 0.57 e Å^−^^3^
653 parameters	Δρ_min_ = −0.52 e Å^−^^3^
Primary atom site location: structure-invariant direct methods	Extinction correction: none

### Special details

**Table:**

Experimental. The low-temperature data was collected with the Oxford Cyrosystem Cobra low-temperature attachment
Geometry. All e.s.d.'s (except the e.s.d. in the dihedral angle between two l.s. planes) are estimated using the full covariance matrix. The cell e.s.d.'s are taken into account individually in the estimation of e.s.d.'s in distances, angles and torsion angles; correlations between e.s.d.'s in cell parameters are only used when they are defined by crystal symmetry. An approximate (isotropic) treatment of cell e.s.d.'s is used for estimating e.s.d.'s involving l.s. planes.
Refinement. Refinement of *F*^2^ against ALL reflections. The weighted *R*-factor *wR* and goodness of fit *S* are based on *F*^2^, conventional *R*-factors *R* are based on *F*, with *F* set to zero for negative *F*^2^. The threshold expression of *F*^2^ > σ(*F*^2^) is used only for calculating *R*-factors(gt) *etc*. and is not relevant to the choice of reflections for refinement. *R*-factors based on *F*^2^ are statistically about twice as large as those based on *F*, and *R*- factors based on ALL data will be even larger.

### Fractional atomic coordinates and isotropic or equivalent isotropic displacement parameters (Å^2^)

**Table:**

	*x*	*y*	*z*	*U*_iso_*/*U*_eq_	
Mn1A	0.34120 (5)	0.29619 (4)	0.11581 (4)	0.01967 (16)	
Cl1A	0.14711 (8)	0.25826 (7)	0.05064 (6)	0.0277 (2)	
O1A	0.3338 (2)	0.41678 (17)	0.18009 (16)	0.0239 (6)	
O2A	0.3473 (2)	0.23461 (17)	0.22530 (16)	0.0221 (6)	
O1WA	0.5284 (2)	0.32040 (19)	0.15261 (17)	0.0298 (7)	
H1WA	0.5748	0.3629	0.1852	0.045*	
H2WA	0.5569	0.2691	0.1424	0.045*	
N1A	0.3597 (2)	0.3558 (2)	0.0007 (2)	0.0202 (7)	
N2A	0.3661 (2)	0.1774 (2)	0.0413 (2)	0.0205 (7)	
C1A	0.3384 (3)	0.5060 (3)	0.1543 (2)	0.0201 (8)	
C2A	0.3191 (3)	0.5846 (3)	0.2162 (3)	0.0229 (9)	
C3A	0.3332 (3)	0.6771 (3)	0.1890 (3)	0.0255 (9)	
H3AA	0.3241	0.7295	0.2300	0.031*	
C4A	0.3601 (3)	0.6964 (3)	0.1044 (3)	0.0262 (9)	
H4AA	0.3688	0.7601	0.0896	0.031*	
C5A	0.3735 (3)	0.6213 (3)	0.0433 (3)	0.0235 (9)	
H5AA	0.3901	0.6336	−0.0142	0.028*	
C6A	0.3624 (3)	0.5244 (2)	0.0661 (2)	0.0194 (8)	
C7A	0.3706 (3)	0.4493 (3)	−0.0049 (2)	0.0211 (8)	
H7AA	0.3851	0.4691	−0.0609	0.025*	
C8A	0.3653 (3)	0.2870 (3)	−0.0760 (2)	0.0206 (8)	
C9A	0.3658 (3)	0.3080 (3)	−0.1665 (3)	0.0263 (9)	
H9AA	0.3611	0.3713	−0.1816	0.032*	
C10A	0.3733 (3)	0.2344 (3)	−0.2347 (3)	0.0288 (10)	
H10A	0.3737	0.2483	−0.2957	0.035*	
C11A	0.3802 (3)	0.1400 (3)	−0.2124 (3)	0.0287 (10)	
H11A	0.3860	0.0912	−0.2585	0.034*	
C12A	0.3787 (3)	0.1177 (3)	−0.1231 (3)	0.0248 (9)	
H12A	0.3830	0.0541	−0.1090	0.030*	
C13A	0.3707 (3)	0.1906 (3)	−0.0536 (3)	0.0220 (9)	
C14A	0.3772 (3)	0.0939 (3)	0.0743 (3)	0.0231 (9)	
H14A	0.3829	0.0415	0.0317	0.028*	
C15A	0.3814 (3)	0.0753 (3)	0.1691 (3)	0.0224 (9)	
C16A	0.3978 (3)	−0.0201 (3)	0.1902 (3)	0.0253 (9)	
H16A	0.4005	−0.0681	0.1423	0.030*	
C17A	0.4095 (3)	−0.0422 (3)	0.2800 (3)	0.0272 (9)	
H17A	0.4169	−0.1057	0.2933	0.033*	
C18A	0.4104 (3)	0.0318 (3)	0.3524 (3)	0.0245 (9)	
H18A	0.4216	0.0161	0.4137	0.029*	
C19A	0.3957 (3)	0.1268 (3)	0.3376 (3)	0.0217 (9)	
C20A	0.3739 (3)	0.1474 (3)	0.2431 (3)	0.0218 (9)	
C21A	0.2845 (4)	0.5662 (3)	0.3085 (3)	0.0331 (11)	
C22A	0.2646 (4)	0.6604 (3)	0.3618 (3)	0.0457 (13)	
H22A	0.2109	0.6893	0.3241	0.069*	
H22B	0.3275	0.7048	0.3756	0.069*	
H22C	0.2429	0.6461	0.4190	0.069*	
C23A	0.1827 (4)	0.4977 (3)	0.2886 (3)	0.0502 (14)	
H23A	0.1294	0.5274	0.2512	0.075*	
H23B	0.1617	0.4851	0.3466	0.075*	
H23C	0.1930	0.4375	0.2556	0.075*	
C24A	0.3691 (4)	0.5217 (3)	0.3717 (3)	0.0461 (13)	
H24A	0.3477	0.5127	0.4303	0.069*	
H24B	0.4331	0.5645	0.3826	0.069*	
H24C	0.3795	0.4598	0.3419	0.069*	
C25A	0.4097 (3)	0.2082 (3)	0.4191 (3)	0.0285 (10)	
C26A	0.3079 (3)	0.2515 (3)	0.4249 (3)	0.0389 (11)	
H26A	0.2885	0.2850	0.3709	0.058*	
H26B	0.3174	0.2965	0.4804	0.058*	
H26C	0.2539	0.2002	0.4270	0.058*	
C27A	0.4468 (4)	0.1702 (3)	0.5141 (3)	0.0396 (12)	
H27A	0.5095	0.1404	0.5121	0.059*	
H27B	0.3936	0.1231	0.5264	0.059*	
H27C	0.4603	0.2236	0.5628	0.059*	
C28A	0.4928 (3)	0.2884 (3)	0.4073 (3)	0.0309 (10)	
H28A	0.4711	0.3157	0.3500	0.046*	
H28B	0.5573	0.2617	0.4057	0.046*	
H28C	0.5022	0.3385	0.4589	0.046*	
Mn1B	0.83334 (4)	0.18866 (4)	0.15141 (4)	0.01899 (16)	
Cl1B	0.64003 (7)	0.14689 (6)	0.08462 (6)	0.0245 (2)	
O1B	0.8253 (2)	0.31322 (17)	0.21049 (16)	0.0249 (6)	
O2B	0.83043 (19)	0.12841 (17)	0.26015 (16)	0.0214 (6)	
O3B	1.0116 (2)	0.2132 (2)	0.19280 (19)	0.0402 (8)	
H1O3	1.0449	0.2199	0.1368	0.060*	
N1B	0.8598 (2)	0.2431 (2)	0.03600 (19)	0.0209 (7)	
N2B	0.8582 (2)	0.0659 (2)	0.0806 (2)	0.0197 (7)	
C1B	0.8462 (3)	0.4012 (3)	0.1851 (3)	0.0222 (9)	
C2B	0.8448 (3)	0.4853 (3)	0.2486 (2)	0.0217 (9)	
C3B	0.8641 (3)	0.5759 (3)	0.2182 (3)	0.0279 (10)	
H3BA	0.8629	0.6309	0.2591	0.034*	
C4B	0.8853 (3)	0.5891 (3)	0.1302 (3)	0.0297 (10)	
H4BA	0.8967	0.6514	0.1126	0.036*	
C5B	0.8889 (3)	0.5093 (3)	0.0698 (3)	0.0270 (9)	
H5BA	0.9038	0.5171	0.0108	0.032*	
C6B	0.8703 (3)	0.4152 (3)	0.0963 (3)	0.0248 (9)	
C7B	0.8741 (3)	0.3358 (3)	0.0270 (3)	0.0228 (9)	
H7BA	0.8884	0.3523	−0.0301	0.027*	
C8B	0.8655 (3)	0.1710 (3)	−0.0386 (2)	0.0218 (9)	
C9B	0.8699 (3)	0.1896 (3)	−0.1297 (2)	0.0249 (9)	
H9BA	0.8695	0.2527	−0.1462	0.030*	
C10B	0.8751 (3)	0.1127 (3)	−0.1958 (3)	0.0277 (9)	
H10B	0.8784	0.1244	−0.2570	0.033*	
C11B	0.8753 (3)	0.0185 (3)	−0.1711 (3)	0.0269 (9)	
H11B	0.8788	−0.0326	−0.2158	0.032*	
C12B	0.8703 (3)	0.0002 (3)	−0.0815 (3)	0.0260 (9)	
H12B	0.8705	−0.0632	−0.0656	0.031*	
C13B	0.8650 (3)	0.0761 (3)	−0.0138 (2)	0.0215 (8)	
C14B	0.8714 (3)	−0.0154 (3)	0.1169 (3)	0.0234 (9)	
H14B	0.8847	−0.0674	0.0777	0.028*	
C15B	0.8675 (3)	−0.0319 (3)	0.2107 (2)	0.0203 (8)	
C16B	0.8852 (3)	−0.1259 (3)	0.2357 (3)	0.0254 (9)	
H16B	0.8978	−0.1728	0.1906	0.030*	
C17B	0.8843 (3)	−0.1491 (3)	0.3238 (3)	0.0284 (10)	
H17B	0.8978	−0.2107	0.3394	0.034*	
C18B	0.8628 (3)	−0.0794 (3)	0.3906 (3)	0.0255 (9)	
H18B	0.8616	−0.0964	0.4506	0.031*	
C19B	0.8432 (3)	0.0140 (3)	0.3720 (2)	0.0204 (8)	
C20B	0.8468 (3)	0.0393 (3)	0.2798 (2)	0.0194 (8)	
C21B	0.8251 (3)	0.4751 (3)	0.3487 (3)	0.0272 (9)	
C22B	0.8269 (4)	0.5748 (3)	0.4038 (3)	0.0345 (11)	
H22D	0.8943	0.6111	0.4094	0.052*	
H22E	0.8128	0.5659	0.4651	0.052*	
H22F	0.7746	0.6099	0.3716	0.052*	
C23B	0.7180 (3)	0.4184 (3)	0.3464 (3)	0.0389 (11)	
H23D	0.7068	0.4140	0.4092	0.058*	
H23E	0.7155	0.3540	0.3153	0.058*	
H23F	0.6645	0.4515	0.3134	0.058*	
C24B	0.9116 (4)	0.4233 (3)	0.3996 (3)	0.0334 (10)	
H24D	0.9777	0.4613	0.4031	0.050*	
H24E	0.9118	0.3605	0.3663	0.050*	
H24F	0.8997	0.4154	0.4617	0.050*	
C25B	0.8180 (3)	0.0874 (3)	0.4473 (2)	0.0252 (9)	
C26B	0.7086 (3)	0.1171 (3)	0.4154 (3)	0.0377 (11)	
H26D	0.6577	0.0610	0.4087	0.057*	
H26E	0.7055	0.1432	0.3565	0.057*	
H26F	0.6943	0.1655	0.4612	0.057*	
C27B	0.8188 (3)	0.0454 (3)	0.5401 (3)	0.0343 (10)	
H27D	0.8874	0.0294	0.5635	0.051*	
H27E	0.7699	−0.0124	0.5310	0.051*	
H27F	0.7996	0.0926	0.5843	0.051*	
C28B	0.9001 (4)	0.1775 (3)	0.4649 (3)	0.0349 (11)	
H28D	0.9684	0.1576	0.4815	0.052*	
H28E	0.8868	0.2213	0.5151	0.052*	
H28F	0.8964	0.2099	0.4093	0.052*	
C29B	1.0736 (4)	0.2242 (4)	0.2779 (4)	0.0556 (14)	
H29D	1.1444	0.2187	0.2709	0.083*	
H29E	1.0512	0.1745	0.3141	0.083*	
H29F	1.0694	0.2871	0.3093	0.083*	

### Atomic displacement parameters (Å^2^)

**Table:**

	*U*^11^	*U*^22^	*U*^33^	*U*^12^	*U*^13^	*U*^23^
Mn1A	0.0292 (4)	0.0130 (3)	0.0194 (3)	0.0058 (2)	0.0105 (3)	0.0002 (2)
Cl1A	0.0274 (6)	0.0307 (5)	0.0277 (5)	0.0065 (4)	0.0099 (4)	0.0058 (4)
O1A	0.0405 (17)	0.0142 (13)	0.0208 (14)	0.0083 (12)	0.0123 (12)	0.0027 (10)
O2A	0.0324 (16)	0.0143 (13)	0.0245 (14)	0.0098 (11)	0.0134 (12)	0.0053 (10)
O1WA	0.0301 (17)	0.0258 (15)	0.0343 (16)	0.0080 (13)	0.0078 (13)	−0.0012 (12)
N1A	0.0219 (18)	0.0177 (16)	0.0211 (17)	0.0047 (14)	0.0058 (14)	−0.0031 (13)
N2A	0.0212 (18)	0.0168 (16)	0.0235 (18)	0.0052 (14)	0.0042 (14)	−0.0009 (13)
C1A	0.025 (2)	0.0156 (19)	0.020 (2)	0.0034 (17)	0.0042 (17)	0.0052 (15)
C2A	0.030 (2)	0.0141 (19)	0.025 (2)	0.0035 (17)	0.0058 (18)	0.0002 (15)
C3A	0.030 (2)	0.0161 (19)	0.030 (2)	0.0054 (17)	0.0018 (18)	0.0000 (16)
C4A	0.026 (2)	0.018 (2)	0.033 (2)	0.0007 (17)	0.0018 (19)	0.0044 (17)
C5A	0.024 (2)	0.021 (2)	0.025 (2)	−0.0020 (17)	0.0033 (17)	0.0070 (16)
C6A	0.021 (2)	0.0137 (18)	0.024 (2)	0.0017 (16)	0.0062 (17)	0.0022 (15)
C7A	0.020 (2)	0.024 (2)	0.021 (2)	0.0033 (17)	0.0069 (17)	0.0048 (16)
C8A	0.021 (2)	0.024 (2)	0.017 (2)	0.0049 (17)	0.0055 (16)	−0.0023 (15)
C9A	0.028 (2)	0.025 (2)	0.026 (2)	0.0044 (18)	0.0079 (18)	−0.0030 (17)
C10A	0.031 (3)	0.039 (2)	0.017 (2)	0.006 (2)	0.0059 (18)	−0.0020 (17)
C11A	0.021 (2)	0.034 (2)	0.030 (2)	0.0060 (19)	0.0063 (18)	−0.0088 (18)
C12A	0.025 (2)	0.025 (2)	0.025 (2)	0.0049 (18)	0.0060 (18)	−0.0009 (17)
C13A	0.016 (2)	0.023 (2)	0.028 (2)	0.0049 (17)	0.0080 (17)	−0.0031 (16)
C14A	0.022 (2)	0.018 (2)	0.028 (2)	0.0051 (17)	0.0056 (18)	−0.0066 (16)
C15A	0.020 (2)	0.0170 (19)	0.031 (2)	0.0039 (16)	0.0075 (18)	−0.0004 (16)
C16A	0.028 (2)	0.0150 (19)	0.032 (2)	0.0043 (17)	0.0058 (18)	−0.0043 (16)
C17A	0.034 (3)	0.0130 (19)	0.038 (3)	0.0083 (18)	0.011 (2)	0.0081 (17)
C18A	0.028 (2)	0.022 (2)	0.029 (2)	0.0087 (18)	0.0106 (18)	0.0116 (17)
C19A	0.024 (2)	0.020 (2)	0.025 (2)	0.0066 (17)	0.0110 (17)	0.0059 (16)
C20A	0.022 (2)	0.0182 (19)	0.028 (2)	0.0060 (17)	0.0104 (17)	0.0057 (16)
C21A	0.059 (3)	0.020 (2)	0.025 (2)	0.016 (2)	0.017 (2)	−0.0006 (17)
C22A	0.081 (4)	0.029 (2)	0.033 (3)	0.021 (2)	0.022 (2)	0.0010 (19)
C23A	0.075 (4)	0.036 (3)	0.054 (3)	0.012 (3)	0.046 (3)	0.005 (2)
C24A	0.090 (4)	0.031 (2)	0.020 (2)	0.028 (3)	0.007 (2)	0.0015 (18)
C25A	0.043 (3)	0.025 (2)	0.023 (2)	0.0136 (19)	0.0112 (19)	0.0062 (17)
C26A	0.053 (3)	0.034 (2)	0.040 (3)	0.019 (2)	0.029 (2)	0.010 (2)
C27A	0.071 (4)	0.030 (2)	0.026 (2)	0.020 (2)	0.020 (2)	0.0065 (18)
C28A	0.049 (3)	0.022 (2)	0.023 (2)	0.011 (2)	0.006 (2)	0.0033 (17)
Mn1B	0.0254 (4)	0.0158 (3)	0.0171 (3)	0.0053 (2)	0.0075 (3)	−0.0013 (2)
Cl1B	0.0258 (6)	0.0210 (5)	0.0282 (5)	0.0066 (4)	0.0074 (4)	0.0021 (4)
O1B	0.0373 (17)	0.0171 (14)	0.0210 (14)	−0.0019 (12)	0.0118 (12)	−0.0012 (11)
O2B	0.0307 (16)	0.0181 (13)	0.0176 (13)	0.0090 (12)	0.0082 (11)	0.0005 (10)
O3B	0.0304 (18)	0.069 (2)	0.0187 (16)	−0.0024 (16)	0.0036 (13)	0.0001 (14)
N1B	0.0265 (19)	0.0211 (17)	0.0161 (16)	0.0058 (14)	0.0076 (14)	−0.0028 (13)
N2B	0.0194 (18)	0.0207 (17)	0.0191 (17)	0.0041 (14)	0.0047 (14)	−0.0015 (13)
C1B	0.023 (2)	0.019 (2)	0.024 (2)	0.0010 (17)	0.0046 (17)	−0.0014 (16)
C2B	0.027 (2)	0.0153 (19)	0.024 (2)	0.0025 (16)	0.0088 (17)	−0.0012 (15)
C3B	0.037 (3)	0.021 (2)	0.024 (2)	0.0052 (19)	0.0039 (19)	−0.0040 (17)
C4B	0.040 (3)	0.016 (2)	0.034 (2)	0.0042 (19)	0.007 (2)	0.0080 (17)
C5B	0.034 (3)	0.026 (2)	0.021 (2)	0.0020 (19)	0.0048 (18)	0.0023 (17)
C6B	0.027 (2)	0.025 (2)	0.020 (2)	−0.0010 (18)	0.0014 (18)	−0.0003 (16)
C7B	0.023 (2)	0.026 (2)	0.019 (2)	0.0024 (18)	0.0062 (17)	0.0028 (16)
C8B	0.021 (2)	0.028 (2)	0.018 (2)	0.0062 (17)	0.0077 (17)	−0.0015 (16)
C9B	0.027 (2)	0.029 (2)	0.020 (2)	0.0083 (18)	0.0069 (18)	0.0028 (16)
C10B	0.025 (2)	0.041 (3)	0.018 (2)	0.0084 (19)	0.0049 (18)	−0.0017 (17)
C11B	0.025 (2)	0.033 (2)	0.021 (2)	0.0058 (18)	0.0051 (18)	−0.0099 (17)
C12B	0.026 (2)	0.024 (2)	0.028 (2)	0.0072 (18)	0.0059 (18)	−0.0048 (17)
C13B	0.018 (2)	0.028 (2)	0.018 (2)	0.0026 (17)	0.0064 (16)	−0.0013 (16)
C14B	0.021 (2)	0.018 (2)	0.031 (2)	0.0064 (17)	0.0071 (18)	−0.0023 (16)
C15B	0.019 (2)	0.0178 (19)	0.025 (2)	0.0032 (16)	0.0065 (17)	−0.0003 (16)
C16B	0.026 (2)	0.017 (2)	0.033 (2)	0.0041 (17)	0.0067 (18)	−0.0017 (17)
C17B	0.030 (3)	0.018 (2)	0.038 (3)	0.0049 (18)	0.007 (2)	0.0050 (18)
C18B	0.027 (2)	0.024 (2)	0.027 (2)	0.0041 (18)	0.0073 (18)	0.0035 (17)
C19B	0.022 (2)	0.022 (2)	0.017 (2)	0.0048 (17)	0.0032 (16)	0.0009 (15)
C20B	0.018 (2)	0.0187 (19)	0.022 (2)	0.0065 (16)	0.0043 (16)	0.0003 (15)
C21B	0.040 (3)	0.018 (2)	0.023 (2)	0.0020 (18)	0.0077 (19)	−0.0047 (16)
C22B	0.054 (3)	0.023 (2)	0.027 (2)	0.004 (2)	0.014 (2)	−0.0037 (17)
C23B	0.045 (3)	0.037 (3)	0.036 (3)	−0.001 (2)	0.021 (2)	−0.008 (2)
C24B	0.052 (3)	0.025 (2)	0.023 (2)	0.006 (2)	0.007 (2)	−0.0022 (17)
C25B	0.033 (3)	0.027 (2)	0.018 (2)	0.0117 (18)	0.0055 (18)	0.0000 (16)
C26B	0.045 (3)	0.047 (3)	0.028 (2)	0.028 (2)	0.012 (2)	0.003 (2)
C27B	0.047 (3)	0.035 (2)	0.024 (2)	0.012 (2)	0.010 (2)	0.0031 (18)
C28B	0.058 (3)	0.022 (2)	0.023 (2)	0.010 (2)	0.001 (2)	−0.0042 (17)
C29B	0.049 (3)	0.057 (3)	0.063 (4)	0.003 (3)	0.021 (3)	−0.002 (3)

### Geometric parameters (Å, °)

**Table:**

Mn1A—O1A	1.863 (2)	Mn1B—N1B	1.978 (3)
Mn1A—O2A	1.874 (2)	Mn1B—N2B	1.997 (3)
Mn1A—N2A	1.979 (3)	Mn1B—O3B	2.293 (3)
Mn1A—N1A	1.981 (3)	Mn1B—Cl1B	2.5416 (11)
Mn1A—O1WA	2.402 (3)	O1B—C1B	1.325 (4)
Mn1A—Cl1A	2.5420 (12)	O2B—C20B	1.325 (4)
O1A—C1A	1.325 (4)	O3B—C29B	1.349 (5)
O2A—C20A	1.327 (4)	O3B—H1O3	0.9992
O1WA—H1WA	0.8500	N1B—C7B	1.305 (5)
O1WA—H2WA	0.8500	N1B—C8B	1.429 (4)
N1A—C7A	1.303 (4)	N2B—C14B	1.303 (4)
N1A—C8A	1.421 (4)	N2B—C13B	1.416 (4)
N2A—C14A	1.306 (4)	C1B—C6B	1.412 (5)
N2A—C13A	1.425 (5)	C1B—C2B	1.429 (5)
C1A—C6A	1.418 (5)	C2B—C3B	1.385 (5)
C1A—C2A	1.423 (5)	C2B—C21B	1.544 (5)
C2A—C3A	1.383 (5)	C3B—C4B	1.385 (5)
C2A—C21A	1.531 (5)	C3B—H3BA	0.9300
C3A—C4A	1.384 (5)	C4B—C5B	1.361 (5)
C3A—H3AA	0.9300	C4B—H4BA	0.9300
C4A—C5A	1.355 (5)	C5B—C6B	1.406 (5)
C4A—H4AA	0.9300	C5B—H5BA	0.9300
C5A—C6A	1.414 (5)	C6B—C7B	1.439 (5)
C5A—H5AA	0.9300	C7B—H7BA	0.9300
C6A—C7A	1.429 (5)	C8B—C9B	1.388 (5)
C7A—H7AA	0.9300	C8B—C13B	1.398 (5)
C8A—C9A	1.380 (5)	C9B—C10B	1.388 (5)
C8A—C13A	1.412 (5)	C9B—H9BA	0.9300
C9A—C10A	1.384 (5)	C10B—C11B	1.389 (5)
C9A—H9AA	0.9300	C10B—H10B	0.9300
C10A—C11A	1.385 (6)	C11B—C12B	1.367 (5)
C10A—H10A	0.9300	C11B—H11B	0.9300
C11A—C12A	1.370 (5)	C12B—C13B	1.394 (5)
C11A—H11A	0.9300	C12B—H12B	0.9300
C12A—C13A	1.391 (5)	C14B—C15B	1.418 (5)
C12A—H12A	0.9300	C14B—H14B	0.9300
C14A—C15A	1.426 (5)	C15B—C16B	1.416 (5)
C14A—H14A	0.9300	C15B—C20B	1.427 (5)
C15A—C16A	1.414 (5)	C16B—C17B	1.356 (5)
C15A—C20A	1.428 (5)	C16B—H16B	0.9300
C16A—C17A	1.361 (5)	C17B—C18B	1.395 (5)
C16A—H16A	0.9300	C17B—H17B	0.9300
C17A—C18A	1.405 (5)	C18B—C19B	1.387 (5)
C17A—H17A	0.9300	C18B—H18B	0.9300
C18A—C19A	1.382 (5)	C19B—C20B	1.430 (5)
C18A—H18A	0.9300	C19B—C25B	1.531 (5)
C19A—C20A	1.421 (5)	C21B—C24B	1.524 (6)
C19A—C25A	1.540 (5)	C21B—C22B	1.533 (5)
C21A—C23A	1.528 (6)	C21B—C23B	1.535 (6)
C21A—C22A	1.528 (5)	C22B—H22D	0.9600
C21A—C24A	1.532 (6)	C22B—H22E	0.9600
C22A—H22A	0.9600	C22B—H22F	0.9600
C22A—H22B	0.9600	C23B—H23D	0.9600
C22A—H22C	0.9600	C23B—H23E	0.9600
C23A—H23A	0.9600	C23B—H23F	0.9600
C23A—H23B	0.9600	C24B—H24D	0.9600
C23A—H23C	0.9600	C24B—H24E	0.9600
C24A—H24A	0.9600	C24B—H24F	0.9600
C24A—H24B	0.9600	C25B—C27B	1.522 (5)
C24A—H24C	0.9600	C25B—C26B	1.537 (5)
C25A—C28A	1.522 (5)	C25B—C28B	1.540 (6)
C25A—C26A	1.528 (5)	C26B—H26D	0.9600
C25A—C27A	1.545 (5)	C26B—H26E	0.9600
C26A—H26A	0.9600	C26B—H26F	0.9600
C26A—H26B	0.9600	C27B—H27D	0.9600
C26A—H26C	0.9600	C27B—H27E	0.9600
C27A—H27A	0.9600	C27B—H27F	0.9600
C27A—H27B	0.9600	C28B—H28D	0.9600
C27A—H27C	0.9600	C28B—H28E	0.9600
C28A—H28A	0.9600	C28B—H28F	0.9600
C28A—H28B	0.9600	C29B—H29D	0.9600
C28A—H28C	0.9600	C29B—H29E	0.9600
Mn1B—O2B	1.865 (2)	C29B—H29F	0.9600
Mn1B—O1B	1.882 (2)		
			
O1A—Mn1A—O2A	91.85 (10)	O2B—Mn1B—N2B	92.16 (11)
O1A—Mn1A—N2A	171.88 (12)	O1B—Mn1B—N2B	171.75 (11)
O2A—Mn1A—N2A	92.91 (11)	N1B—Mn1B—N2B	81.67 (12)
O1A—Mn1A—N1A	92.10 (11)	O2B—Mn1B—O3B	88.18 (11)
O2A—Mn1A—N1A	170.09 (12)	O1B—Mn1B—O3B	89.86 (11)
N2A—Mn1A—N1A	82.19 (12)	N1B—Mn1B—O3B	83.42 (11)
O1A—Mn1A—O1WA	90.91 (10)	N2B—Mn1B—O3B	84.42 (11)
O2A—Mn1A—O1WA	86.48 (10)	O2B—Mn1B—Cl1B	95.77 (8)
N2A—Mn1A—O1WA	82.82 (11)	O1B—Mn1B—Cl1B	97.12 (9)
N1A—Mn1A—O1WA	84.37 (11)	N1B—Mn1B—Cl1B	91.90 (9)
O1A—Mn1A—Cl1A	96.40 (9)	N2B—Mn1B—Cl1B	88.18 (9)
O2A—Mn1A—Cl1A	99.22 (8)	O3B—Mn1B—Cl1B	171.74 (8)
N2A—Mn1A—Cl1A	89.33 (9)	C1B—O1B—Mn1B	131.2 (2)
N1A—Mn1A—Cl1A	89.37 (9)	C20B—O2B—Mn1B	131.5 (2)
O1WA—Mn1A—Cl1A	170.55 (7)	C29B—O3B—Mn1B	130.4 (3)
C1A—O1A—Mn1A	132.0 (2)	C29B—O3B—H1O3	118.3
C20A—O2A—Mn1A	130.3 (2)	Mn1B—O3B—H1O3	111.2
Mn1A—O1WA—H1WA	136.5	C7B—N1B—C8B	121.3 (3)
Mn1A—O1WA—H2WA	113.8	C7B—N1B—Mn1B	124.7 (2)
H1WA—O1WA—H2WA	107.7	C8B—N1B—Mn1B	114.0 (2)
C7A—N1A—C8A	121.9 (3)	C14B—N2B—C13B	122.2 (3)
C7A—N1A—Mn1A	124.2 (2)	C14B—N2B—Mn1B	124.5 (2)
C8A—N1A—Mn1A	113.8 (2)	C13B—N2B—Mn1B	113.3 (2)
C14A—N2A—C13A	122.1 (3)	O1B—C1B—C6B	122.1 (3)
C14A—N2A—Mn1A	124.2 (2)	O1B—C1B—C2B	119.7 (3)
C13A—N2A—Mn1A	113.7 (2)	C6B—C1B—C2B	118.2 (3)
O1A—C1A—C6A	121.0 (3)	C3B—C2B—C1B	117.8 (3)
O1A—C1A—C2A	119.5 (3)	C3B—C2B—C21B	121.4 (3)
C6A—C1A—C2A	119.5 (3)	C1B—C2B—C21B	120.8 (3)
C3A—C2A—C1A	117.0 (3)	C4B—C3B—C2B	123.7 (3)
C3A—C2A—C21A	122.2 (3)	C4B—C3B—H3BA	118.2
C1A—C2A—C21A	120.8 (3)	C2B—C3B—H3BA	118.2
C2A—C3A—C4A	123.9 (4)	C5B—C4B—C3B	118.9 (4)
C2A—C3A—H3AA	118.0	C5B—C4B—H4BA	120.5
C4A—C3A—H3AA	118.0	C3B—C4B—H4BA	120.5
C5A—C4A—C3A	119.1 (4)	C4B—C5B—C6B	120.3 (4)
C5A—C4A—H4AA	120.4	C4B—C5B—H5BA	119.8
C3A—C4A—H4AA	120.4	C6B—C5B—H5BA	119.8
C4A—C5A—C6A	120.7 (3)	C5B—C6B—C1B	121.1 (3)
C4A—C5A—H5AA	119.6	C5B—C6B—C7B	116.0 (3)
C6A—C5A—H5AA	119.6	C1B—C6B—C7B	122.9 (3)
C5A—C6A—C1A	119.5 (3)	N1B—C7B—C6B	126.8 (3)
C5A—C6A—C7A	116.9 (3)	N1B—C7B—H7BA	116.6
C1A—C6A—C7A	123.5 (3)	C6B—C7B—H7BA	116.6
N1A—C7A—C6A	126.6 (3)	C9B—C8B—C13B	120.6 (3)
N1A—C7A—H7AA	116.7	C9B—C8B—N1B	125.0 (3)
C6A—C7A—H7AA	116.7	C13B—C8B—N1B	114.5 (3)
C9A—C8A—C13A	120.0 (3)	C10B—C9B—C8B	119.2 (4)
C9A—C8A—N1A	125.4 (3)	C10B—C9B—H9BA	120.4
C13A—C8A—N1A	114.6 (3)	C8B—C9B—H9BA	120.4
C8A—C9A—C10A	119.7 (4)	C9B—C10B—C11B	120.3 (3)
C8A—C9A—H9AA	120.2	C9B—C10B—H10B	119.9
C10A—C9A—H9AA	120.2	C11B—C10B—H10B	119.9
C9A—C10A—C11A	120.3 (4)	C12B—C11B—C10B	120.5 (3)
C9A—C10A—H10A	119.9	C12B—C11B—H11B	119.8
C11A—C10A—H10A	119.9	C10B—C11B—H11B	119.8
C12A—C11A—C10A	120.8 (4)	C11B—C12B—C13B	120.4 (4)
C12A—C11A—H11A	119.6	C11B—C12B—H12B	119.8
C10A—C11A—H11A	119.6	C13B—C12B—H12B	119.8
C11A—C12A—C13A	119.8 (4)	C12B—C13B—C8B	119.1 (3)
C11A—C12A—H12A	120.1	C12B—C13B—N2B	125.4 (3)
C13A—C12A—H12A	120.1	C8B—C13B—N2B	115.5 (3)
C12A—C13A—C8A	119.4 (3)	N2B—C14B—C15B	126.1 (3)
C12A—C13A—N2A	125.8 (3)	N2B—C14B—H14B	117.0
C8A—C13A—N2A	114.9 (3)	C15B—C14B—H14B	117.0
N2A—C14A—C15A	126.3 (3)	C16B—C15B—C14B	116.7 (3)
N2A—C14A—H14A	116.9	C16B—C15B—C20B	119.0 (3)
C15A—C14A—H14A	116.9	C14B—C15B—C20B	124.3 (3)
C16A—C15A—C14A	116.9 (3)	C17B—C16B—C15B	121.5 (3)
C16A—C15A—C20A	119.1 (3)	C17B—C16B—H16B	119.3
C14A—C15A—C20A	124.0 (3)	C15B—C16B—H16B	119.3
C17A—C16A—C15A	120.5 (3)	C16B—C17B—C18B	119.2 (4)
C17A—C16A—H16A	119.7	C16B—C17B—H17B	120.4
C15A—C16A—H16A	119.7	C18B—C17B—H17B	120.4
C16A—C17A—C18A	119.3 (3)	C19B—C18B—C17B	123.2 (3)
C16A—C17A—H17A	120.3	C19B—C18B—H18B	118.4
C18A—C17A—H17A	120.3	C17B—C18B—H18B	118.4
C19A—C18A—C17A	123.5 (3)	C18B—C19B—C20B	117.6 (3)
C19A—C18A—H18A	118.3	C18B—C19B—C25B	121.3 (3)
C17A—C18A—H18A	118.3	C20B—C19B—C25B	121.1 (3)
C18A—C19A—C20A	116.9 (3)	O2B—C20B—C15B	121.3 (3)
C18A—C19A—C25A	121.8 (3)	O2B—C20B—C19B	119.2 (3)
C20A—C19A—C25A	121.2 (3)	C15B—C20B—C19B	119.5 (3)
O2A—C20A—C19A	118.8 (3)	C24B—C21B—C22B	108.1 (3)
O2A—C20A—C15A	121.1 (3)	C24B—C21B—C23B	110.6 (3)
C19A—C20A—C15A	120.1 (3)	C22B—C21B—C23B	107.3 (3)
C23A—C21A—C22A	107.6 (4)	C24B—C21B—C2B	108.5 (3)
C23A—C21A—C2A	109.5 (3)	C22B—C21B—C2B	111.6 (3)
C22A—C21A—C2A	111.7 (3)	C23B—C21B—C2B	110.9 (3)
C23A—C21A—C24A	110.1 (4)	C21B—C22B—H22D	109.5
C22A—C21A—C24A	107.7 (3)	C21B—C22B—H22E	109.5
C2A—C21A—C24A	110.2 (3)	H22D—C22B—H22E	109.5
C21A—C22A—H22A	109.5	C21B—C22B—H22F	109.5
C21A—C22A—H22B	109.5	H22D—C22B—H22F	109.5
H22A—C22A—H22B	109.5	H22E—C22B—H22F	109.5
C21A—C22A—H22C	109.5	C21B—C23B—H23D	109.5
H22A—C22A—H22C	109.5	C21B—C23B—H23E	109.5
H22B—C22A—H22C	109.5	H23D—C23B—H23E	109.5
C21A—C23A—H23A	109.5	C21B—C23B—H23F	109.5
C21A—C23A—H23B	109.5	H23D—C23B—H23F	109.5
H23A—C23A—H23B	109.5	H23E—C23B—H23F	109.5
C21A—C23A—H23C	109.5	C21B—C24B—H24D	109.5
H23A—C23A—H23C	109.5	C21B—C24B—H24E	109.5
H23B—C23A—H23C	109.5	H24D—C24B—H24E	109.5
C21A—C24A—H24A	109.5	C21B—C24B—H24F	109.5
C21A—C24A—H24B	109.5	H24D—C24B—H24F	109.5
H24A—C24A—H24B	109.5	H24E—C24B—H24F	109.5
C21A—C24A—H24C	109.5	C27B—C25B—C19B	112.2 (3)
H24A—C24A—H24C	109.5	C27B—C25B—C26B	107.5 (3)
H24B—C24A—H24C	109.5	C19B—C25B—C26B	109.5 (3)
C28A—C25A—C26A	109.4 (3)	C27B—C25B—C28B	107.4 (3)
C28A—C25A—C19A	109.7 (3)	C19B—C25B—C28B	109.8 (3)
C26A—C25A—C19A	111.8 (3)	C26B—C25B—C28B	110.3 (3)
C28A—C25A—C27A	107.0 (3)	C25B—C26B—H26D	109.5
C26A—C25A—C27A	107.3 (3)	C25B—C26B—H26E	109.5
C19A—C25A—C27A	111.5 (3)	H26D—C26B—H26E	109.5
C25A—C26A—H26A	109.5	C25B—C26B—H26F	109.5
C25A—C26A—H26B	109.5	H26D—C26B—H26F	109.5
H26A—C26A—H26B	109.5	H26E—C26B—H26F	109.5
C25A—C26A—H26C	109.5	C25B—C27B—H27D	109.5
H26A—C26A—H26C	109.5	C25B—C27B—H27E	109.5
H26B—C26A—H26C	109.5	H27D—C27B—H27E	109.5
C25A—C27A—H27A	109.5	C25B—C27B—H27F	109.5
C25A—C27A—H27B	109.5	H27D—C27B—H27F	109.5
H27A—C27A—H27B	109.5	H27E—C27B—H27F	109.5
C25A—C27A—H27C	109.5	C25B—C28B—H28D	109.5
H27A—C27A—H27C	109.5	C25B—C28B—H28E	109.5
H27B—C27A—H27C	109.5	H28D—C28B—H28E	109.5
C25A—C28A—H28A	109.5	C25B—C28B—H28F	109.5
C25A—C28A—H28B	109.5	H28D—C28B—H28F	109.5
H28A—C28A—H28B	109.5	H28E—C28B—H28F	109.5
C25A—C28A—H28C	109.5	O3B—C29B—H29D	109.5
H28A—C28A—H28C	109.5	O3B—C29B—H29E	109.5
H28B—C28A—H28C	109.5	H29D—C29B—H29E	109.5
O2B—Mn1B—O1B	93.60 (10)	O3B—C29B—H29F	109.5
O2B—Mn1B—N1B	170.00 (11)	H29D—C29B—H29F	109.5
O1B—Mn1B—N1B	91.79 (11)	H29E—C29B—H29F	109.5
			
O2A—Mn1A—O1A—C1A	171.6 (3)	O1B—Mn1B—O2B—C20B	−169.9 (3)
N1A—Mn1A—O1A—C1A	0.7 (3)	N1B—Mn1B—O2B—C20B	−47.5 (8)
O1WA—Mn1A—O1A—C1A	85.1 (3)	N2B—Mn1B—O2B—C20B	4.1 (3)
Cl1A—Mn1A—O1A—C1A	−88.9 (3)	O3B—Mn1B—O2B—C20B	−80.2 (3)
O1A—Mn1A—O2A—C20A	−165.3 (3)	Cl1B—Mn1B—O2B—C20B	92.5 (3)
N2A—Mn1A—O2A—C20A	8.2 (3)	O2B—Mn1B—O3B—C29B	−28.2 (4)
O1WA—Mn1A—O2A—C20A	−74.5 (3)	O1B—Mn1B—O3B—C29B	65.4 (4)
Cl1A—Mn1A—O2A—C20A	98.0 (3)	N1B—Mn1B—O3B—C29B	157.3 (4)
O1A—Mn1A—N1A—C7A	5.4 (3)	N2B—Mn1B—O3B—C29B	−120.5 (4)
N2A—Mn1A—N1A—C7A	−168.8 (3)	O2B—Mn1B—N1B—C7B	−116.6 (6)
O1WA—Mn1A—N1A—C7A	−85.3 (3)	O1B—Mn1B—N1B—C7B	6.0 (3)
Cl1A—Mn1A—N1A—C7A	101.8 (3)	N2B—Mn1B—N1B—C7B	−168.9 (3)
O1A—Mn1A—N1A—C8A	−177.4 (2)	O3B—Mn1B—N1B—C7B	−83.6 (3)
N2A—Mn1A—N1A—C8A	8.4 (2)	Cl1B—Mn1B—N1B—C7B	103.2 (3)
O1WA—Mn1A—N1A—C8A	91.9 (2)	O2B—Mn1B—N1B—C8B	61.4 (8)
Cl1A—Mn1A—N1A—C8A	−81.0 (2)	O1B—Mn1B—N1B—C8B	−176.0 (3)
O2A—Mn1A—N2A—C14A	1.6 (3)	N2B—Mn1B—N1B—C8B	9.1 (2)
N1A—Mn1A—N2A—C14A	172.9 (3)	O3B—Mn1B—N1B—C8B	94.4 (3)
O1WA—Mn1A—N2A—C14A	87.7 (3)	Cl1B—Mn1B—N1B—C8B	−78.8 (2)
Cl1A—Mn1A—N2A—C14A	−97.6 (3)	O2B—Mn1B—N2B—C14B	−3.6 (3)
O2A—Mn1A—N2A—C13A	−178.6 (2)	N1B—Mn1B—N2B—C14B	168.5 (3)
N1A—Mn1A—N2A—C13A	−7.2 (2)	O3B—Mn1B—N2B—C14B	84.3 (3)
O1WA—Mn1A—N2A—C13A	−92.5 (2)	Cl1B—Mn1B—N2B—C14B	−99.3 (3)
Cl1A—Mn1A—N2A—C13A	82.2 (2)	O2B—Mn1B—N2B—C13B	179.1 (2)
Mn1A—O1A—C1A—C6A	−6.9 (5)	N1B—Mn1B—N2B—C13B	−8.8 (2)
Mn1A—O1A—C1A—C2A	173.2 (3)	O3B—Mn1B—N2B—C13B	−93.0 (2)
O1A—C1A—C2A—C3A	174.9 (3)	Cl1B—Mn1B—N2B—C13B	83.3 (2)
C6A—C1A—C2A—C3A	−5.0 (5)	Mn1B—O1B—C1B—C6B	7.2 (6)
O1A—C1A—C2A—C21A	−5.1 (6)	Mn1B—O1B—C1B—C2B	−172.8 (3)
C6A—C1A—C2A—C21A	175.0 (4)	O1B—C1B—C2B—C3B	−178.1 (3)
C1A—C2A—C3A—C4A	2.9 (6)	C6B—C1B—C2B—C3B	1.9 (6)
C21A—C2A—C3A—C4A	−177.1 (4)	O1B—C1B—C2B—C21B	3.4 (6)
C2A—C3A—C4A—C5A	0.2 (6)	C6B—C1B—C2B—C21B	−176.6 (3)
C3A—C4A—C5A—C6A	−1.3 (6)	C1B—C2B—C3B—C4B	−0.4 (6)
C4A—C5A—C6A—C1A	−0.8 (6)	C21B—C2B—C3B—C4B	178.1 (4)
C4A—C5A—C6A—C7A	175.7 (3)	C2B—C3B—C4B—C5B	−0.9 (6)
O1A—C1A—C6A—C5A	−175.8 (3)	C3B—C4B—C5B—C6B	0.8 (6)
C2A—C1A—C6A—C5A	4.0 (5)	C4B—C5B—C6B—C1B	0.7 (6)
O1A—C1A—C6A—C7A	7.9 (6)	C4B—C5B—C6B—C7B	179.0 (4)
C2A—C1A—C6A—C7A	−172.2 (4)	O1B—C1B—C6B—C5B	177.9 (4)
C8A—N1A—C7A—C6A	177.3 (3)	C2B—C1B—C6B—C5B	−2.1 (6)
Mn1A—N1A—C7A—C6A	−5.7 (5)	O1B—C1B—C6B—C7B	−0.2 (6)
C5A—C6A—C7A—N1A	−177.8 (4)	C2B—C1B—C6B—C7B	179.9 (4)
C1A—C6A—C7A—N1A	−1.4 (6)	C8B—N1B—C7B—C6B	179.9 (4)
C7A—N1A—C8A—C9A	−10.7 (6)	Mn1B—N1B—C7B—C6B	−2.3 (6)
Mn1A—N1A—C8A—C9A	172.0 (3)	C5B—C6B—C7B—N1B	179.8 (4)
C7A—N1A—C8A—C13A	169.2 (3)	C1B—C6B—C7B—N1B	−2.1 (6)
Mn1A—N1A—C8A—C13A	−8.1 (4)	C7B—N1B—C8B—C9B	−10.6 (6)
C13A—C8A—C9A—C10A	−1.0 (6)	Mn1B—N1B—C8B—C9B	171.3 (3)
N1A—C8A—C9A—C10A	178.8 (4)	C7B—N1B—C8B—C13B	170.3 (3)
C8A—C9A—C10A—C11A	0.1 (6)	Mn1B—N1B—C8B—C13B	−7.8 (4)
C9A—C10A—C11A—C12A	0.7 (6)	C13B—C8B—C9B—C10B	−0.8 (6)
C10A—C11A—C12A—C13A	−0.4 (6)	N1B—C8B—C9B—C10B	−179.8 (4)
C11A—C12A—C13A—C8A	−0.6 (5)	C8B—C9B—C10B—C11B	0.3 (6)
C11A—C12A—C13A—N2A	178.6 (3)	C9B—C10B—C11B—C12B	0.1 (6)
C9A—C8A—C13A—C12A	1.3 (5)	C10B—C11B—C12B—C13B	0.0 (6)
N1A—C8A—C13A—C12A	−178.6 (3)	C11B—C12B—C13B—C8B	−0.5 (6)
C9A—C8A—C13A—N2A	−178.0 (3)	C11B—C12B—C13B—N2B	179.2 (3)
N1A—C8A—C13A—N2A	2.1 (5)	C9B—C8B—C13B—C12B	0.8 (6)
C14A—N2A—C13A—C12A	5.4 (6)	N1B—C8B—C13B—C12B	180.0 (3)
Mn1A—N2A—C13A—C12A	−174.4 (3)	C9B—C8B—C13B—N2B	−178.8 (3)
C14A—N2A—C13A—C8A	−175.3 (3)	N1B—C8B—C13B—N2B	0.4 (5)
Mn1A—N2A—C13A—C8A	4.9 (4)	C14B—N2B—C13B—C12B	10.1 (6)
C13A—N2A—C14A—C15A	175.1 (3)	Mn1B—N2B—C13B—C12B	−172.5 (3)
Mn1A—N2A—C14A—C15A	−5.1 (5)	C14B—N2B—C13B—C8B	−170.3 (3)
N2A—C14A—C15A—C16A	−177.7 (4)	Mn1B—N2B—C13B—C8B	7.1 (4)
N2A—C14A—C15A—C20A	0.0 (6)	C13B—N2B—C14B—C15B	179.1 (3)
C14A—C15A—C16A—C17A	175.7 (4)	Mn1B—N2B—C14B—C15B	2.0 (5)
C20A—C15A—C16A—C17A	−2.1 (6)	N2B—C14B—C15B—C16B	−179.4 (4)
C15A—C16A—C17A—C18A	−3.2 (6)	N2B—C14B—C15B—C20B	0.8 (6)
C16A—C17A—C18A—C19A	2.7 (6)	C14B—C15B—C16B—C17B	179.3 (4)
C17A—C18A—C19A—C20A	3.0 (6)	C20B—C15B—C16B—C17B	−0.8 (6)
C17A—C18A—C19A—C25A	−172.9 (4)	C15B—C16B—C17B—C18B	1.6 (6)
Mn1A—O2A—C20A—C19A	165.3 (3)	C16B—C17B—C18B—C19B	−0.8 (6)
Mn1A—O2A—C20A—C15A	−14.3 (5)	C17B—C18B—C19B—C20B	−0.8 (6)
C18A—C19A—C20A—O2A	172.2 (3)	C17B—C18B—C19B—C25B	178.9 (4)
C25A—C19A—C20A—O2A	−11.8 (5)	Mn1B—O2B—C20B—C15B	−2.8 (5)
C18A—C19A—C20A—C15A	−8.2 (5)	Mn1B—O2B—C20B—C19B	177.6 (2)
C25A—C19A—C20A—C15A	167.7 (3)	C16B—C15B—C20B—O2B	179.5 (3)
C16A—C15A—C20A—O2A	−172.5 (3)	C14B—C15B—C20B—O2B	−0.6 (6)
C14A—C15A—C20A—O2A	9.8 (6)	C16B—C15B—C20B—C19B	−0.8 (5)
C16A—C15A—C20A—C19A	7.9 (5)	C14B—C15B—C20B—C19B	179.1 (3)
C14A—C15A—C20A—C19A	−169.7 (3)	C18B—C19B—C20B—O2B	−178.8 (3)
C3A—C2A—C21A—C23A	121.2 (4)	C25B—C19B—C20B—O2B	1.5 (5)
C1A—C2A—C21A—C23A	−58.8 (5)	C18B—C19B—C20B—C15B	1.6 (5)
C3A—C2A—C21A—C22A	2.1 (6)	C25B—C19B—C20B—C15B	−178.1 (3)
C1A—C2A—C21A—C22A	−177.9 (4)	C3B—C2B—C21B—C24B	−117.2 (4)
C3A—C2A—C21A—C24A	−117.5 (4)	C1B—C2B—C21B—C24B	61.2 (5)
C1A—C2A—C21A—C24A	62.5 (5)	C3B—C2B—C21B—C22B	1.7 (5)
C18A—C19A—C25A—C28A	121.5 (4)	C1B—C2B—C21B—C22B	−179.9 (4)
C20A—C19A—C25A—C28A	−54.2 (5)	C3B—C2B—C21B—C23B	121.2 (4)
C18A—C19A—C25A—C26A	−117.0 (4)	C1B—C2B—C21B—C23B	−60.4 (5)
C20A—C19A—C25A—C26A	67.3 (5)	C18B—C19B—C25B—C27B	0.6 (5)
C18A—C19A—C25A—C27A	3.1 (5)	C20B—C19B—C25B—C27B	−179.8 (3)
C20A—C19A—C25A—C27A	−172.6 (3)	C18B—C19B—C25B—C26B	−118.8 (4)
O2B—Mn1B—O1B—C1B	162.8 (3)	C20B—C19B—C25B—C26B	60.9 (5)
N1B—Mn1B—O1B—C1B	−8.8 (3)	C18B—C19B—C25B—C28B	119.9 (4)
O3B—Mn1B—O1B—C1B	74.6 (3)	C20B—C19B—C25B—C28B	−60.4 (4)
Cl1B—Mn1B—O1B—C1B	−100.9 (3)		

### Hydrogen-bond geometry (Å, °)

**Table:**

*D*—H···*A*	*D*—H	H···*A*	*D*···*A*	*D*—H···*A*
O1WA—H2WA···Cl1B	0.85	2.28	3.113 (3)	167
O3B—H1O3···Cl1A^i^	1.00	2.06	3.026 (3)	163
C5A—H5AA···O1WA^ii^	0.93	2.55	3.463 (5)	169
C4B—H4BA···Cl1A^ii^	0.93	2.79	3.528 (4)	137
C12B—H12B···Cl1A^iii^	0.93	2.73	3.646 (4)	170
C23A—H23C···O1A	0.96	2.34	2.984 (6)	124
C23B—H23E···O1B	0.96	2.35	2.983 (5)	123
C24A—H24C···O1A	0.96	2.34	2.975 (5)	123
C24B—H24E···O1B	0.96	2.36	3.010 (5)	124
C26A—H26A···O2A	0.96	2.45	3.041 (5)	119
C26B—H26E···O2B	0.96	2.35	2.998 (5)	124
C28A—H28A···O2A	0.96	2.34	2.977 (5)	124
C28B—H28F···O2B	0.96	2.34	2.968 (5)	122
C14B—H14B···Cg1^iv^	0.93	3.23	3.690 (4)	113

Symmetry codes:  (i) *x*+1, *y*, *z*; (ii) −*x*+1, −*y*+1, −*z*; (iii) −*x*+1, −*y*, −*z*; (iv) −*x*+2, −*y*, −*z*.
